# Serum total bilirubin levels are negatively correlated with metabolic syndrome in aged Chinese women: a community-based study

**DOI:** 10.1590/1414-431X20165252

**Published:** 2017-01-26

**Authors:** P. Zhong, D.M. Sun, D.H. Wu, T.M. Li, X.Y. Liu, H.Y. Liu

**Affiliations:** 1 Department of Neurology, Shanghai TCM Integrated Hospital, Shanghai University of Chinese Medicine, Shanghai, China; 2 Puxing Community Health Service Centers, Pudong New Area, Shanghai, China; 3 Department of Neurology, the Third People's Hospital, Shanghai Jiao Tong University School of Medicine, Shanghai, China; 4 Department of Neurology, Shanghai Fifth People’s Hospital, Fudan University, Shanghai, China; 5 Pingliang Community Health Service Centers, Yangpu Area, Shanghai, China

**Keywords:** Total bilirubin, Metabolic syndrome, Oxidative stress

## Abstract

We evaluated serum total bilirubin levels as a predictor for metabolic syndrome (MetS) and investigated the relationship between serum total bilirubin levels and MetS prevalence. This cross-sectional study included 1728 participants over 65 years of age from Eastern China. Anthropometric data, lifestyle information, and previous medical history were collected. We then measured serum levels of fasting blood-glucose, total cholesterol, triglycerides, and total bilirubin, as well as alanine aminotransferase activity. The prevalence of MetS and each of its individual component were calculated per quartile of total bilirubin level. Logistic regression was used to assess the correlation between serum total bilirubin levels and MetS. Total bilirubin level in the women who did not have MetS was significantly higher than in those who had MetS (P<0.001). Serum total bilirubin quartiles were linearly and negatively correlated with MetS prevalence and hypertriglyceridemia (HTG) in females (P<0.005). Logistic regression showed that serum total bilirubin was an independent predictor of MetS for females (OR: 0.910, 95%CI: 0.863–0.960; P=0.001). The present study suggests that physiological levels of serum total bilirubin might be an independent risk factor for aged Chinese women, and the prevalence of MetS and HTG are negatively correlated to serum total bilirubin levels.

## Introduction

Metabolic syndrome (MetS) is a group of common, complex disorders including insulin resistance, hypertension, dyslipidemia, and obesity (especially abdominal obesity) (1). It is also strongly associated with cardiovascular disease. The overall prevalence of MetS in adult populations is about 20–30%, depending on age, ethnicity, gender, and diagnostic criteria (2,3). The incidence of MetS has been shown to increase with age (2), and aged populations are therefore at high risk for MetS.

The exact pathophysiological characteristics of MetS are still unclear. However, studies show that systemic inflammation, insulin resistance, altered adipokine levels and oxidative stress play important roles in MetS development (4,5). Meanwhile, bilirubin has been characterized as a potent antioxidant (6–8) and has been demonstrated to be negatively associated with oxidative stress (9,10). Several studies have also shown that bilirubin levels are negatively related to hypertension, diabetes (11,12), MetS, and insulin resistance (13). A retrospective study in 6205 male Koreans showed that bilirubin levels are negatively correlated with the incidence of MetS (14), and could be used as a predictor for MetS. Other studies have also shown that the serum bilirubin level can be used as an early biomarker for the progression to MetS in asymptomatic patients (15). Together, these findings suggest that bilirubin plays a role in the development of MetS.

All studies to date investigating the association between bilirubin and MetS focus on young people (16), a wide range of ages, or postmenopausal women. Importantly, the association has not been examined in aged people. In the present cross-sectional study, people of 65 years of age or older from 11 communities in Eastern China were included. We evaluated serum total bilirubin levels as a predictor for MetS, and further analyzed the potential relationship between serum total bilirubin levels and MetS prevalence.

## Subjects and Methods

### Subjects

Participants in this cross-sectional study were males or females ≥65 years of age that lived in any of the 11 communities in Pudong New Area, Shanghai, for at least 5 years. Enrollment took place between January–March 2012. Exclusion criteria were clinical or laboratory evidence of severe systemic diseases (such as cancer, renal failure, or heart failure), goiter, liver dysfunction (activities of alanine aminotransferase higher than 3-fold the upper limit of the normal range (120 U/L); or total bilirubin level higher than 17.1 μmol/L), or HIV-AIDS. In addition, athletes (including professional and amateur athletes) and participants who regularly exercised were also excluded.

General information was obtained, including date of birth, physical activity, and history of smoking and drinking. Previous medical history was obtained with a standardized questionnaire. Participants who smoked every day or every few days, and had smoked more than 100 cigarettes during their lifetime were considered smokers. Daily alcohol consumption was converted into grams of alcohol consumed per day. Male participants consuming more than 20–30 g of alcohol per day or female participants consuming more than 10–20 g of alcohol per day were considered drinkers. Participants with a known history of using oral antidiabetic agents or insulin were considered diabetic. Participants with a history of acute myocardial infarction, or coronary angiogram showing stenosis of the coronary artery ≥50% were considered to have a history of coronary heart disease. Participants with systolic blood pressure ≥140 mmHg, diastolic blood pressure ≥90 mmHg, or with a history of using oral anti-hypertensive drugs were considered to have a history of hypertension.

This study was approved by the Institutional Review Board of the Third People's Hospital, Shanghai Jiaotong University School of Medicine, and performed according to the Helsinki Declaration. All participants signed informed consents.

### Anthropometric data

Participant anthropometric data were collected by trained medical staff following standard procedures. Blood pressure was measured twice after 10 min of rest, and the mean was calculated for analyses. Body mass index (BMI) was calculated as weight/height^2^ (kg/m^2^).

### Biochemical data

Participants were asked to fast for at least 8 h. Blood was collected and placed in room temperature for 1-3 h and was then centrifuged at 1704 *g* for 15 min to separate the serum. Levels of fasting blood-glucose (FBG), total cholesterol (TC), triglycerides (TG), and total bilirubin (TBIL), as well as alanine aminotransferase (ALT) activity were measured in an automatic biochemical analyzer (Hitachi 7600-020, Japan) using enzyme colorimetric method with reagent from Roche company (Switzerland).

### Definition of MetS

MetS was diagnosed according to the Chinese Diabetes Society criteria (2004) (17). Participants with three or more of the following items were diagnosed with MetS: 1) hypertension: blood pressure ≥140/90 mmHg, or use of anti-hypertensive medication; 2) hypertriglycerides: fasting plasma triglycerides ≥1.7 mmol/L, or low high-density lipoprotein level (HDL; <1 mmol/L for males and <1.3 mmol/L for females); 3) hyperglycemia: FBG ≥6.1 mmol/L, or use of anti-diabetic agents; and 4) overweight or obesity: BMI ≥25 kg/m^2^.

### Statistical analyses

SPSS v.13.0 software (USA) was used for statistical analyses. Quantitative data with normal distributions are reported as means and standard deviations (SD). Quantitative data with skewed distributions are reported as quartiles, and qualitative data are reported as rates. Analysis of variance was used for comparing quantitative data between three or more groups, while non-parametric tests were used for comparisons of qualitative data. Participants were divided into quartiles according to total bilirubin levels as follows: Q1, <10.1 μmol/L for males and <8.8 μmol/L for females; Q2, 10.1–12.15 μmol/L for males and 8.8–10.7 μmol/L for females; Q3, 12.15–14.4 μmol/L for males and 10.7–12.7 μmol/L for females, and Q4, ≥14.4 μmol/L for males and ≥12.7 μmol/L for females. The prevalence of MetS and of each component was then estimated. Logistic regression was used to assess the independent predictors of MetS in males and females. P<0.05 was considered to be statistically significant.

## Results

### Baseline characteristics

Baseline characteristics of participants are summarized in Table 1. A total of 1728 eligible participants including 744 males (43.1%) and 984 females (56.9%) were enrolled in the present study. The mean age of the participants was 72.68±6.33 and 73.49±6.87 years for males and females, respectively. This difference was statistically significant (P=0.011). The prevalence of MetS was 25.3 and 30.1% in the male and female subjects, respectively, and the difference was statistically significant (P<0.001). Similarly, the proportions of diabetes, coronary heart disease, and hypertension in the male and female subjects were significantly different. BMI and FBG were not significantly different between the two genders. However, weight, height, blood pressure, TG, TC, ALT and TBIL were significantly different between the two genders (P<0.05).

**Table:**
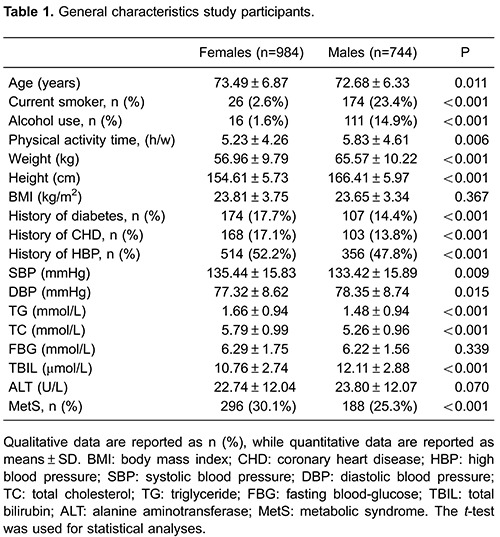

### Characteristics of female participants

The prevalence of MetS in female subjects was 30.1%. The clinical and biochemical characteristics of the females are shown in Table 2. The levels of some components of the MetS (including the FBG, blood pressure, TG, and BMI) were significantly higher, while the total bilirubin level was significantly lower in females with MetS than in those without MetS (P=0.002).

**Table:**
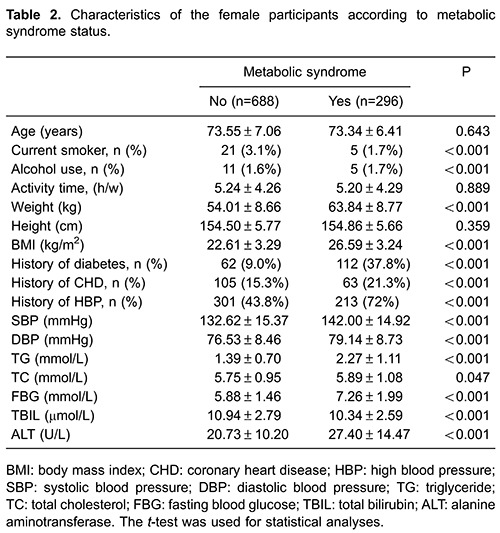

### Associations between total bilirubin level and the risk of MetS

The prevalence of MetS and its components in the sample are summarized in Table 3. The number of males in each quartile of total bilirubin level was as follows: Q1: 187, Q2: 185, Q3: 189, and Q4: 183, and the mean ages were 72.57±6.14, 72.72±6.39, 72.68±6.47, and 72.75±6.37 years, respectively. For females, the number of participants in each quartile was as follows: Q1: 258, Q2: 240, Q3: 249, and Q4: 237, and the mean ages were 74.46±7.01, 72.65±6.38, 73.28±7.21, and 74.49±6.72 years, respectively. The prevalence of MetS and hypertriglyceridemia (HTG) decreased with increased levels of total bilirubin, and a linear correlation was found between the quartiles of total bilirubin levels and the prevalence of MetS and HTG.

**Table:**
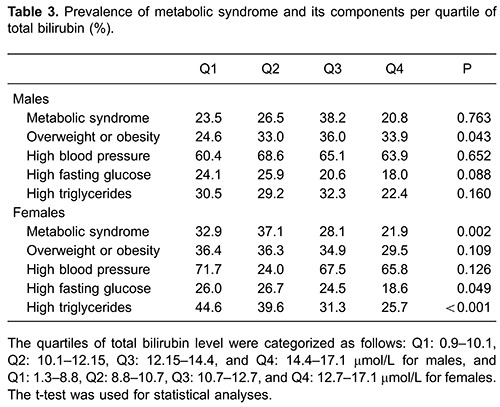

### Independent predictors of MetS

Logistic regression was performed with MetS prevalence as the dependent variable. Independent variables were age, gender, physical activity, smoking, drinking, total bilirubin, total cholesterol and ALT. We found that total bilirubin (OR: 0.952, 95%CI: 0.915–0.990; P=0.014) and ALT (OR: 1.048, 95%CI: 1.038–1.058; P<0.001) were independent predictors of MetS. Results of the stratified analyses by gender are shown in Table 4. These findings suggest that every 1 μmol/L increase in total bilirubin level reduces the risk of female MetS by 9.0% (OR: 0.910, 95%CI: 0.863–0.960; P=0.001).

**Table:**
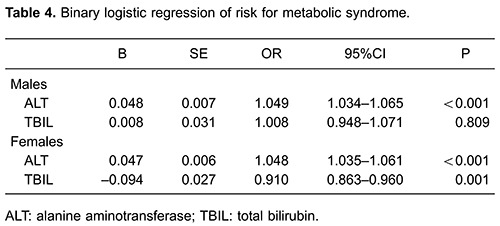

## Discussion

In the present cross-sectional study in aged patients from Eastern China, we found that serum total bilirubin may be an independent risk factor for MetS in females. We also found that the prevalence of MetS and HTG were negatively related to the serum levels of total bilirubin.

Association between serum total bilirubin levels and MetS risk is still under debate. In another study performed in Shanghai that included participants over 40 years of age, Wu et al. (18) showed that serum total bilirubin levels were negatively associated with MetS risk in both females and males. In contrast, a Japanese study found that serum total bilirubin levels were negatively associated with the risk of MetS in males but not in females (19). However, the participants included in the Japanese study were over 20 years of age, which may explain this discrepancy. Another study in the USA found that serum total bilirubin levels were negatively associated with MetS, as well as with central obesity and low HDL levels, in participants between the ages of 12 and 17 years. However, no comparison between males and females was performed (16). Bilirubin levels may be affected by several factors including diet and smoking (20). However, this hypothesis could not be verified since no detailed information was available in the present study. Future studies should be performed to investigate whether the correlation between the total bilirubin level and MetS is associated with the status of smoking, drinking, and diet.

The molecular mechanisms involved in the protective effects of bilirubin on MetS are still unclear. Recent studies show that oxidative stress plays an important role in insulin resistance and the development of MetS (5). *In vitro* and *in vivo* evidence demonstrate that bilirubin is an endogenous antioxidant, which may inhibit the oxidation of lipid and lipoprotein under physiological conditions (6,21). It is estimated that bilirubin accounts for approximately 10% of all antioxidant effects in healthy adults, and the level of bilirubin is negatively associated with serum levels of oxidative biomarkers (22,23). Increased levels of bilirubin may therefore result in higher antioxidant effects, which in turn reduce the risk of developing MetS.

We also show that total serum bilirubin levels are negatively correlated with HTG and high FGB in aged females. These findings agree with other studies showing that total bilirubin serum levels are closely linked to triglyceride levels, hypertension, diabetes, and insulin resistance, which are risk factors for MetS (14,24). Another study shows that physiological levels of serum total bilirubin were negatively associated with MetS in a Korean sample without distinct metabolic or cardiovascular disorders. The study further revealed a negative relationship between total bilirubin and abdominal obesity as well as with HTG (25). Other reports have shown that bilirubin may function as an endogenous lipid-reducing agent, thereby affecting lipid metabolism (26). This pathological mechanism underlies Gilbert syndrome, in which an increase in indirect bilirubin levels enhance cholesterol excretion and decrease its production, thereby disrupting lipid balance (26). However, in the present study, only total bilirubin was measured. Thus, association between total serum bilirubin levels and MetS risk could not be further assessed to differentiate the effects of indirect and direct bilirubin.

Our findings should be considered in light of a few limitations. First, as a cross-sectional study, we could not conclusively show a causal relationship between the serum total bilirubin levels and MetS in females. Second, the effects from other underlying diseases, as well as drugs for diabetes, coronary heart disease, hypertension, and hyperlipidemia could not be clearly elucidated. Total bilirubin level and ALT activity were measured only once for each patient, yet they may have transient fluctuations; therefore, not all the underlying liver diseases could be clearly diagnosed by the data collected. Third, the data about smoking and drinking may not be very correct, as these data were self-reported. Fourth, we only measured total bilirubin level, while the direct and indirect bilirubin levels were not measured.

In summary, this large community-based study suggests that a physiological level of serum total bilirubin is an independent risk factor for aged Chinese women. Furthermore, we found that the prevalence of MetS and HTG was negatively related to physiological levels of serum total bilirubin.

## References

[1] Oda E (2012). Metabolic syndrome: its history, mechanisms, and
limitations. Acta Diabetol.

[2] Ervin RB (2009). Prevalence of metabolic syndrome among adults 20 years of age and
over, by sex, age, race and ethnicity, and body mass index: United States,
2003–2006. Natl Health Stat Report.

[3] Grundy SM (2008). Metabolic syndrome pandemic. Arterioscler Thromb Vasc Biol.

[4] Grundy SM, Cleeman JI, Daniels SR, Donato KA, Eckel RH, Franklin BA (2006). Diagnosis and management of the metabolic syndrome: an American Heart
Association/National Heart, Lung, and Blood Institute scientific
statement. Curr Opin Cardiol.

[5] Roberts CK, Sindhu KK (2009). Oxidative stress and metabolic syndrome. Life Sci.

[6] Tomaro ML, Batlle AM (2002). Bilirubin: its role in cytoprotection against oxidative
stress. Int J Biochem Cell Biol.

[B07] Stocker R, Yamamoto Y, McDonagh AF, Glazer AN, Ames BN (1987). Bilirubin is an antioxidant of possible physiological
importance. Science.

[8] Frei B, Stocker R, Ames BN (1988). Antioxidant defenses and lipid peroxidation in human blood
plasma. Proc Natl Acad Sci U S A.

[9] Vitek L (2012). The role of bilirubin in diabetes, metabolic syndrome, and
cardiovascular diseases. Front Pharmacol.

[10] Erdogan D, Gullu H, Yildirim E, Tok D, Kirbas I, Ciftci O (2006). Low serum bilirubin levels are independently and inversely related to
impaired flow-mediated vasodilation and increased carotid intima-media thickness
in both men and women. Atherosclerosis.

[11] Perlstein TS, Pande RL, Beckman JA, Creager MA (2008). Serum total bilirubin level and prevalent lower-extremity peripheral
arterial disease: National Health and Nutrition Examination Survey (NHANES) 1999
to 2004. Arterioscler Thromb Vasc Biol.

[12] Perlstein TS, Pande RL, Creager MA, Weuve J, Beckman JA (2008). Serum total bilirubin level, prevalent stroke, and stroke outcomes:
NHANES 1999–2004. Am J Med.

[13] Guzek M, Jakubowski Z, Bandosz P, Wyrzykowski B, Smoczynski M, Jabloiska A (2012). Inverse association of serum bilirubin with metabolic syndrome and
insulin resistance in Polish population. Przegl Epidemiol.

[14] Lee MJ, Jung CH, Kang YM, Hwang JY, Jang JE, Leem J (2014). Serum bilirubin as a predictor of incident metabolic syndrome: a
4-year retrospective longitudinal study of 6205 initially healthy Korean
men. Diabetes Metab.

[15] Jenko-Pražnikar Z, Petelin A, Jurdana M, Žiberna L (2013). Serum bilirubin levels are lower in overweight asymptomatic
middle-aged adults: An early indicator of metabolic syndrome?. Metabolism.

[16] Lin LY, Kuo HK, Hwang JJ, Lai LP, Chiang FT, Tseng CD (2009). Serum bilirubin is inversely associated with insulin resistance and
metabolic syndrome among children and adolescents. Atherosclerosis.

[17] Alberti KG, Eckel RH, Grundy SM, Zimmet PZ, Cleeman JI, Donato KA (2009). Harmonizing the metabolic syndrome: a joint interim statement of the
International Diabetes Federation Task Force on Epidemiology and Prevention;
National Heart, Lung, and Blood Institute; American Heart Association; World Heart
Federation; International Atherosclerosis Society; and International Association
for the Study of Obesity. Circulation.

[18] Wu Y, Li M, Xu M, Bi Y, Li X, Chen Y (2011). Low serum total bilirubin concentrations are associated with increased
prevalence of metabolic syndrome in Chinese. J Diabetes.

[19] Ishizaka N, Ishizaka Y, Toda E, Nagai R, Yamakado M (2005). Association between serum uric acid, metabolic syndrome, and carotid
atherosclerosis in Japanese individuals. Arterioscler Thromb Vasc Biol.

[20] Schwertner HA (1998). Association of smoking and low serum bilirubin antioxidant
concentrations. Atherosclerosis.

[21] Liu Y, Liu J, Tetzlaff W, Paty DW, Cynader MS (2006). Biliverdin reductase, a major physiologic cytoprotectant, suppresses
experimental autoimmune encephalomyelitis. Free Radic Biol Med.

[22] Kalousova M, Novotny L, Zima T, Braun M, Vitek L (2005). Decreased levels of advanced glycation end-products in patients with
Gilbert syndrome. Cell Mol Biol.

[23] Demir M, Demir C, Cosar S (2013). The relationship between serum bilirubin concentration and coronary
slow flow. Ther Adv Cardiovasc Dis.

[24] Song YS, Koo BK, Cho NH, Moon MK (2014). Effect of low serum total bilirubin levels (≤0.32 mg/dL) on Risk of
coronary artery disease in patients with metabolic syndrome. Am J Cardiol.

[25] Choi SH, Yun KE, Choi HJ (2013). Relationships between serum total bilirubin levels and metabolic
syndrome in Korean adults. Nutr Metab Cardiovasc Dis.

[26] Bulmer AC, Verkade HJ, Wagner KH (2013). Bilirubin and beyond: a review of lipid status in Gilbert's syndrome
and its relevance to cardiovascular disease protection. Prog Lipid Res.

